# Environmental correlates of physical activity among children 10 to 13 years old in Wallonia (Belgium)

**DOI:** 10.1186/s12889-019-6509-7

**Published:** 2019-02-13

**Authors:** Camille Pedroni, Maud Dujeu, Nathalie Moreau, Thérésa Lebacq, Estelle Méroc, Isabelle Godin, Katia Castetbon

**Affiliations:** 1 Service d’Information Promotion Éducation Santé (SIPES), Research Center in Epidemiology, Biostatistics and Clinical Research, School of Public Health, Université libre de Bruxelles (ULB), Postal address: ULB CP598, Route de Lennik 808, B-1070 Brussels, Belgium; 2 Research Center in Social Approaches to Health, School of Public Health, Université libre de Bruxelles (ULB), Brussels, Belgium

**Keywords:** Physical activity, Living environment, Children

## Abstract

**Background:**

In Belgium, as in many other countries, the juvenile practice of physical activity is insufficient. A growing attention has been paid to environmental factors that may influence physical activity but with inconsistent findings. This study aims to estimate the association between daily life environment characteristics and physical activity among children 10 to 13 years old in Wallonia (Belgium).

**Methods:**

Data were collected using a self-administered questionnaire among 1940 children (HBSC survey). Associations between factors related to the children’s living environment and physical activity (vigorous physical activity (VPA) ≥ twice a week; global physical activity (GPA) defined as VPA ≥ twice a week and moderate-to-vigorous physical activity ≥1 h/day) were estimated using logistic regressions adjusted for potential confounders.

**Results:**

Nearly three-quarters of the children practiced VPA ≥ twice a week, but only one in five practiced GPA consistent with recommendations. After adjustment, children living in a neighborhood with playgrounds or parks were more likely to achieve a recommended level of GPA (OR: 1.34 [1.04–1.73]), as were children who reported that other youngsters were present in their neighborhood with whom they could play outside (OR: 1.50 [1.12–1.99]). The presence of neighborhood children was also positively associated with VPA (OR: 1.80 [1.42–2.29]); in stratified analyses, the association was significant only among boys (OR: 1.95 [1.34–2.82]). Moreover, and only in girls (OR: 1.66 [1.10–2.49]), a feeling of safety in one’s neighborhood was positively associated with VPA. No association was found between VPA and the existence of a yard or a garden at home to go playing outside.

**Conclusion:**

Our results argue for developing actions aimed at creating living environments more favorable to children’s daily physical activity. More specifically, they help better understand the environment of Belgian children and thus contribute to better identify their needs.

## Background

Regular physical activity during childhood and adolescence provides numerous benefits for immediate and future health [1, 2]. Studies have shown that it helps youth develop a healthy loco-motor system (bones, muscles and joints), good control and coordination of movements and a healthy cardiovascular system (heart and lungs). It may also contribute to appropriate weight and favorable psychological health. The practice of regular physical activity might thus prevent certain chronic diseases that occur later in life [1, 2].

For youngsters 5 to 17 years of age, the World Health Organization (WHO) recommends practicing daily moderate-to-vigorous physical activity (MVPA) for at least 60 min, and vigorous physical activity (VPA) at least three times a week [1]. In Europe, the practice of physical activity is insufficient among adolescent populations, especially among girls [3, 4]. In Belgium, a 2014 national study revealed that only one-third of 10–17-year-olds practiced daily MVPA for at least 60 min [5]. International studies have shown that physical activity decreases with age during adolescence [6]. Moreover, a longitudinal study found that children who often practiced physical activity were significantly more likely to regularly engage in sports and physical activities during adulthood [7]. These results highlight the need for sensitizing children at an early age about the importance of a regular physical activity and for encouraging them to be physically active. To support this process, more studies looking at ways to increase physical activity among children are needed.

Identifying and understanding factors that can improve childhood and adolescent physical activity is necessary to design and implement effective interventions. Recent years have witnessed a growing interest in environmental factors that influence physical activity--physical (e.g. physical structures and facilities), social (e.g. support and norms) and institutional (e.g., school rules and policies). Studying the juvenile environment, mainly the school and home, would help understand what might contribute to increased physical activity. In addition to sports activities, one major contribution to childhood physical activity lies in free active play and unstructured physical activities taking place outdoors during their free time. This type of play provides numerous benefits in terms of cognitive, social and physical development [8–12]. A systematic review published in 2015 found consistent evidence of the contribution of time spent outdoors to childhood physical activity [10]. Other studies have shown that exposure to natural settings may contribute to children’s cognitive functioning and stimulate general learning and motor development [11, 12]. Indeed, important lessons have been learned so far from exploring environmental factors associated with PA and other health and developmental outcomes in children.

Four reviews focusing on environmental correlates of physical activity among children and adolescents [13–16] concluded that the presence of recreational facilities in the neighborhood (e.g., parks, playgrounds) was positively associated with physical activity. Three of the four reviews found that traffic safety was inversely associated with physical activity [14–16]. Recreational facilities and traffic safety are the most studied factors and associations with physical activity are significant and consistent in the literature [14–16]. Nevertheless, other environmental factors such as climate and season, living in a house with a garden, home equipment... have been so far less studied in children and available results are somewhat divergent. Inconsistencies between conclusions might originate to measurement (objective or subjective) of physical activity and to environmental factors addressed, as well as to complex characteristics for some of them [14]. For example, overall “perceived security” is difficult to measure and interpret, since numerous dimensions are incorporated into this term. This has led to divergent conclusions among studies, which may use the global term “perceived security” in their conclusions while, in fact, they have not studied the same dimension (e.g., traffic safety and criminality). Many studies have also focused on a specific type of physical activity (e.g., active commuting to school) or MVPA; relatively few studies have analyzed VPA or global physical activity. Moreover, most studies focusing on this topic were conducted in the United States and Australia, and cannot be directly extrapolated to Europe. Finally, despite some research developed in Belgium but with a different purpose and in other settings [17–20], no study of this nature has been carried out in Wallonia, whereas access to free playgrounds may differ from that in other backgrounds. Further research is thus needed to provide evidence and to explore environmental factors not sufficiently elucidated up until now, such as the presence of other children in the neighborhood or that of a garden/yard at home.

In French-speaking Belgium (Wallonia and Brussels regions), the Health Behavior in School-aged Children (HBSC) survey provides data on physical activity and perception of the surrounding environment of children in fifth and sixth grade elementary education. The aim of the present study was to estimate the association between characteristics of the daily environment and physical activity among children 10 to 13 years old in Wallonia.

## Methods

### Sample

This study is based on data collected for the 2014 HBSC survey conducted in French-speaking schools in Belgium. The HBSC study is a cross-sectional school-based survey undertaken every four years in over 40 countries and regions using an international standardized protocol [21]. In French-speaking Belgium, the protocol was approved by the educational authorities of each school network (private and public).

Self-administered questionnaires were filled out in the classroom according to a standardized procedure, and treated as confidential and anonymous [22]. Students ranging from fifth grade in elementary schools (around 10 years of age) to final grades in secondary schools (18 years in most situations) were asked about their health status, well-being and health-related behavior. Only students in the fifth and sixth grades of elementary education were included in the present analyses, since older students were not asked questions related to their living environment.

A two-stage random sample was used. First, schools were randomly selected from an official list of all schools stratified per province and school network, using an allocation proportional to the school population size of each province and network. Among the 781 schools invited to participate, 168 took part in the survey, with a participation rate of 46% among respondents. Secondly, one class per grade was randomly selected in the participating schools. The sample included all students in the selected classes, who had been present on the day of questionnaire completion. The entire 2014 HBSC sample was composed of 14,122 adolescents. The distribution of adolescent participants by province and school orientation (general, technical or vocational education) was compared to distribution observed in the Wallonia-Brussels Federation (FWB) reference school population. Overall, the sample distribution was very close to that observed in the reference population [23]. A total of 2510 children from fifth and sixth elementary grades in 82 Walloon schools participated in the study.

### Measures

#### Physical activity

The question related to MVPA was: “Over the past seven days, on how many days were you physically active for a total of ≥ 60 minutes per day?” Eight answer options were available, ranging from “never” to “seven days”. The variable was then dichotomized to identify those who met WHO guidelines [1], especially concerning the practice of ≥60 min of MVPA daily. This indicator is an adaptation of the MVPA screening measure, which had been previously validated [24]. This question was adapted by the international HBSC network and has been used in HBSC surveys since 2001.

The question “Outside of school hours: how often do you usually exercise intensely in your free time so much that you become out of breath or sweat?” aimed to assess the level of VPA. Response options were: “never”, “less than once a month”, “once a month”, “1 to 3 times a month”, “once a week”, “2 to 3 times a week”, “4 to 6 times a week” or “every day”. Categories were dichotomized into those practicing VPA ≥ twice a week versus those who did not reach this threshold, which was chosen because it is closest to WHO recommendations.

A composite variable was created on the basis of these two indicators in order to assess the global physical activity (GPA) level of children. This variable separated children into two categories: those who practiced daily MVPA for ≥60 min and VPA ≥ twice a week versus the others.

#### Environmental factors

Four indicators were used. One concerned the perceived security of the neighborhood: “It is safe to walk or play alone in my neighborhood during the day”. The other three concerned physical environmental factors: “There are other children near my home to go and play with outside”; “There is somewhere at home I can go out and play”; and “There are playgrounds or parks close to my home where I can play”. For each indicator, the child reported the extent to which he/she agreed (“I strongly agree”, “I neither agree nor disagree” or “I strongly disagree”). These four indicators are derived from a broader tool aimed at measuring the impact of environmental influences on children’s physical activity. When tested on a large sample of children and adolescents who took part in the European Youth Heart Study, it showed satisfactory validity [25].

#### Covariates

Socio-demographic factors considered were: gender (boys/girls), age (10–11 years/12–13 years), size of the municipality in which the school was located (< 3000 inhabitants/3000 to 15,000 inhabitants/> 15,000 inhabitants), migratory status (children born in Belgium of both parents born in Belgium/children born in Belgium of one or two parent(s) born abroad/children born abroad).

Adolescents were asked to indicate other persons with whom they were living in their predominant household. Based on this question, they were classified into having four types of family structure: “living with both parents”, “stepfamily”, “single-parent family” or “other situation” (e.g., adolescents living in a foster home). Due to co-linearity, the dichotomized variable (living with both parents versus other situations) was used.

In addition, the children’s socio-economic status was assessed using the family affluence scale (FAS), based on six indicators related to common material assets (ownership of a car, child’s own bedroom, number of computers, number of bathrooms, ownership of a dishwasher and frequency of holiday abroad during the last year) [4]. A score, previously validated for Europe [26], has been established by the sum of answers (score’s range: 0 to 13). A “low” FAS corresponded to values from 0 to 6, “medium” FAS was 7 to 9, and “high” FAS was 10 to 13.

### Statistical analyses

Univariate analyses were initially aimed at analyzing factors associated with the practice of a recommended level of GPA and a recommended level of VPA. Pearson’s chi-square test was used. Linear trend analysis was performed using the chi-square test for trend when categories were ordered and proportions increased or decreased according to the order of the categories. The odds ratio (OR) and 95% confidence interval [95%CI] were calculated using logistic regression.

All indicators significantly associated with outcome having a *P*-value < 20% were included in initial multivariate logistic regression models. Non-significant associations were manually removed from models one by one to retain only statistically significant associations in the final models (*P* < 0.05). Confounding was considered when the difference between crude and adjusted OR of other variable(s) was equal to or greater than 10%. In that case, the variable was kept in the model [27]. Interactions with gender were tested in the final models. Conditions of application of logistic regressions were verified. All statistical analyses were performed using Stata® V.14.

## Results

**Fig. 1:**
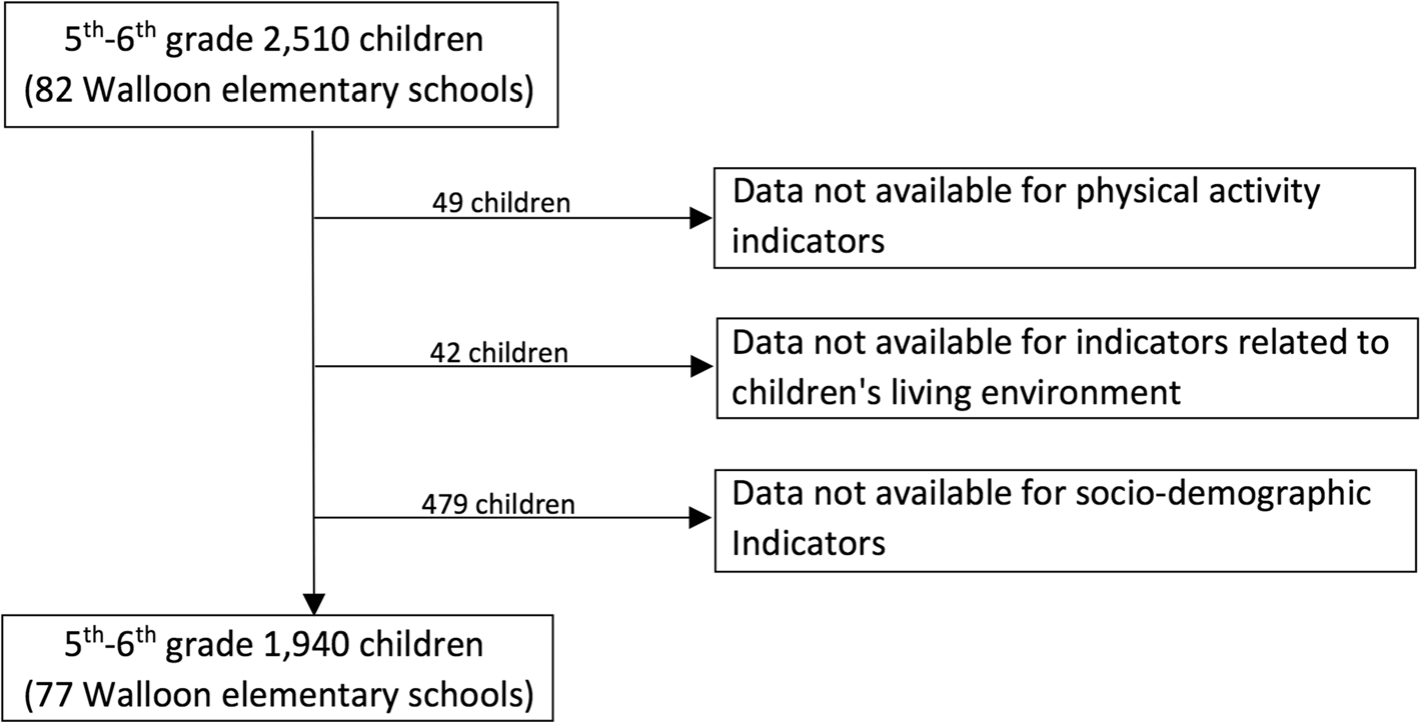
Flow chart of inclusion in analyses

In total, 2510 children in fifth and sixth elementary grades from 82 Walloon schools participated in the study (Fig. 1). The final sample analyzed comprised 1940 children from 77 Walloon elementary schools after removing observations with missing information.

Table 1 presents sample characteristics. Twenty-three percent of children had daily MVPA of ≥60 min, three-quarters practiced VPA ≥ twice a week and only one-fifth met these two thresholds. Most children lived in a house with a garden or yard for playing outside, and 57.2% lived in a neighborhood where there were other children with whom they could play outdoors. Nearly half lived in an area with a playground or park nearby, and they felt their neighborhood was safe for walking and playing alone outside (Table 1).

**Table 1:** Characteristics of the sample (*n* = 1940). HBSC, French-speaking Belgium, 2014

	N	**%**
Gender		
Boys	1004	51.8
Girls	936	48.2
Age		
10–11 years	1397	72.0
12–13 years	543	28.0
Family structure		
Living with both parents	1340	69.1
Other family situations	600	30.9
Family affluence scale		
High	672	34.6
Medium	953	49.1
Low	315	16.2
Size of the area in which the school is located		
< 3000 inhabitants	508	26.2
Between 3000 and 15,000 inhabitants	926	47.7
> 15,000 inhabitants	506	26.1
Migratory status		
Born in Belgium of both parents born in Belgium	1417	73.0
Born in Belgium of one or two parent(s) born abroad	381	19.6
Born abroad	142	7.3
Safe neighborhood for playing or walking alone		
Strongly agree	891	45.9
Neither agree nor disagree	667	34.4
Strongly disagree	382	19.7
Presence of other children in the neighborhood for playing outside		
Strongly agree	1110	57.2
Neither agree nor disagree	331	17.1
Strongly disagree	499	25.7
Presence of a garden/yard at home for outdoor play		
Strongly agree	1726	89.0
Neither agree nor disagree	89	4.6
Strongly disagree	125	6.4
Presence of a playground or a park in the neighborhood		
Strongly agree	904	46.6
Neither agree nor disagree Strongly disagree	321 715	16.6 36.9
Daily moderate-to-vigorous physical activity (MVPA) ≥ 60 min		
No	1488	76.7
Yes	452	23.3
Vigorous physical activity (VPA) ≥ twice a week		
No	470	24.2
Yes	1470	75.8
Sufficient overall physical activity (GPA)^a^		
No	1539	79.3
Yes	401	20.7

^a^Daily practice of a MVPA ≥60 min combined with a VPA ≥ twice a week

### Recommended global physical activity (GPA)

Univariate analysis showed that gender (male vs. female), migratory status (born abroad vs. born in Belgium and both parents born in Belgium), perceived security of the neighborhood (“I strongly agree” vs. “I strongly disagree”), presence of other children (“I strongly agree” vs. “I strongly disagree”) and of a playground or park (“I strongly agree” vs. “I strongly disagree”) were significantly associated with a recommended level of GPA (Table 2).

**Table 2:** Factors associated with the practice of a recommended level of overall physical activity^a^. HBSC, French-speaking Belgium, 2014

	N	%	Crude OR (95%CI)	p	Adjusted OR (95%CI) Complete model	p	Adjusted OR (95%CI) Final model	p
Gender				< 0.001		< 0.001		< 0.001
Boys	1004	26.4	2.11 (1.68–2.65)		1.97 (1.56–2.49)		2.03 (1.61–2.56)	
Girls	936	14.5	1		1		1	
Age				0.33				
10–11 years	1397	20.1	0.89 (0.70–1.13)					
12–13 years	543	22.1	1					
Family structure				0.12		0.18		
Living with both parents	1340	19.7	1		1			
Other family situations	600	22.8	1.21 (0.96–1.52)		1.18 (0.93–1.50)			
Family affluence scale				0.36				
High	672	21.4	1					
Medium	953	19.4	0.88 (0.69–1.13)					
Low	315	22.9	1.09 (0.79–1.50)					
Size of the area in which the school is located				0.20		0.16		
< 3000 inhabitants	508	23.4	1.27 (0.94–1.72)		1.35 (0.98–.186)			
Between 3000 and 15,000 inhabitants	926	19.9	1.03 (0.78–1.36)		1.11 (0.83–1.48)			
> 15,000 inhabitants	506	19.4	1		1			
Migratory status				0.01		0.03		0.04
Born in Belgium of both parents born in Belgium	1417	19.3	1		1		1	
Born in Belgium of one or two parent(s) born abroad	381	22.3	1.20 (0.91–1.58)		1.25 (0.94–1.66)		1.20 (0.90–1.59)	
Born abroad	142	29.6	1.75 (1.20–2.57)		1.67 (1.12–2.50)		1.63 (1.10–2.42)	
Safe neighborhood for walking or playing alone				0.002		0.34		
Strongly agree	891	24.1	1.44 (1.07–1.95)		1.09 (0.79–1.52)			
Neither agree nor disagree	667	17.5	0.96 (0.70–1.34)		0.90 (0.64–1.26)			
Strongly disagree	382	18.1	1		1			
Presence of other children in the neighborhood for playing outdoors				< 0.001		0.006		0.001
Strongly agree	1110	24.4	1.72 (1.30–2.26)		1.44 (1.06–1.94)		1.50 (1.12–1.99)	
Neither agree nor disagree	331	15.4	0.97 (0.66–1.42)		0.91 (0.61–1.35)		0.90 (0.61–1.33)	
Strongly disagree	499	15.8	1		1		1	
Presence of a garden/yard at home for playing outdoors				0.20		0.20		
Strongly agree	1726	20.6	0.75 (0.50–1.14)		0.72 (0.46–1.12)			
Neither agree nor disagree	89	15.7	0.54 (0.27–1.08)		0.54 (0.26–1.11)			
Strongly disagree	125	25.6	1		1			
Presence of a playground/park				0.001		0.04		0.04
Strongly agree	904	24.3	1.53 (1.20–1.96)		1.35 (1.04–1.75)		1.34 (1.04–1.73)	
Neither agree nor disagree	321	17.8	1.03 (0.73–1.45)		1.00 (0.70–1.43)		0.99 (0.70–1.41)	
Strongly disagree	715	17.3	1		1		1	

^a^Daily practice of a MVPA ≥60 min combined with a VPA ≥ twice a week

Observed associations remained statistically significant in the final multivariate model, except for the association with perceived neighborhood security (Table 2). Boys were significantly more likely to achieve GPA recommendations than girls. This was also the case for children born abroad compared to children born in Belgium. Children who strongly agreed with the fact that there was a playground or park in their neighborhood were more likely to achieve a recommended level of GPA than those who strongly disagreed (OR: 1.34 [1.04–1.73]). Similarly, children who had expressed strong agreement with the fact that there were other children in the neighborhood with whom they could play outside were more likely to have a sufficient level of GPA than those who strongly disagreed (OR: 1.50 [1.12–1.99]).

### Recommended vigorous physical activity (VPA)

In univariate analysis, gender (male vs. female), age (10–11 years vs. 12–13 years), family structure (living with both parents vs. other situations), level of family affluence (high level vs. low or medium level), perceived security of the neighborhood (“I strongly agree” vs. “I strongly disagree”) and the presence of other children in the neighborhood (“I strongly agree” vs. “I strongly disagree” or “I neither agree nor disagree”) were significantly associated with the practice of a recommended level of VPA (Table 3). The linear trend test showed that the higher the level of family affluence, the higher the proportion of children with recommended VPA.

**Table 3:** Factors associated with the practice of vigorous physical activity (VPA) at least twice a week. HBSC, French-speaking Belgium, 2014

	N	%	Crude OR (95%CI)	P	Adjusted OR (95%CI) Complete model	p	Adjusted OR (95%CI) Final model	p
Gender				< 0.001		< 0.001		< 0.001
Boys	1004	81.0	1.81 (1.46–2.23)		1.72 (1.38–2.13)		1.75 (1.41–2.17)	
Girls	936	70.2	1		1		1	
Age				0.01		0.06		
10–11 years	1397	77.3	1.34 (1.07–1.67)		1.26 (0.99–1.59)			
12–13 years	543	71.8	1		1			
Family structure				0.001		0.005		0.002
Living with both parents	1340	77.9	1		1		1	
Other family situations	600	71.0	0.769 (0.56–0.86)		0.72 (0.58–0.90)		0.70 (0.56–0.88)	
Family affluence scale				< 0.001^a^		< 0.001		< 0.001
High	672	82.1	1		1		1	
Medium	953	73.8	0.61 (0.48–0.78)		0.66 (0.51–0.85)		0.64 (0.56–0.88)	
Low	315	68.2	0.47 (0.34–0.64)		0.54 (0.40–0.75)		0.52 (0.38–0.71)	
Size of the area where the school is located				0.12		0.74		
< 3000 inhabitants	508	78.9	1.35 (1.01–1.80)		1.12 (0.83–1.51)			
Between 3000 and 15,000 inhabitants	926	75.3	1.10 (0.86–1.40)		1.02 (0.79–1.31)			
> 15,000 inhabitants	506	73.5	1		1			
Migratory status				0.42				
Born in Belgium of both parents born in Belgium	1417	75.5	1					
Born in Belgium of one or two parent(s) born abroad	381	75.1	0.98 (0.75–1.27)					
Born abroad	142	80.3	1.32 (0.86–2.02)					
Safe neighborhood for walking or playing alone				< 0.001		0.35		
Strongly agree	891	79.7	1.69 (1.29–2.22)		1.24 (0.92–1.67)			
Neither agree nor disagree	667	73.9	1.22 (0.92–1.61)		1.09 (0.81–1.45)			
Strongly disagree	382	69.9	1		1			
Presence of other children in the neighborhood for playing outdoors				< 0.001		< 0.001		< 0.001
Strongly agree	1110	79.2	1.93 (1.53-2.44)		1.68 (1.30–2.18)		1.80 (1.42–2.29)	
Neither agree nor disagree	331	78.6	1.86 (1.35–2.56)		1.72 (1.24–2.40)		1.76 (1.27–2.44)	
Strongly disagree	499	66.3	1		1		1	
Presence of a garden/yard at home for playing outdoors				0.63				
Strongly agree	1726	76.1	1.09 (0.72–1.65)					
Neither agree nor disagree	89	71.9	0.88 (0.48–1.62)					
Strongly disagree	125	74.4	1					
Presence of a playground/park				0.31				
Strongly agree	904	77.3	1.19 (0.95–1.50)					
Neither agree nor disagree	321	75.1	1.05 (0.78–1.42)					
Strongly disagree	715	74.1	1					

^a^Linear trend test

Associations with age and perceived security observed in univariate analysis were no longer statistically significant in the multivariate logistic regression model (Table 3). The final model showed that boys (versus girls), children having a “high” level of family affluence (versus those in the “medium” and “low” brackets) and children living with both parents (versus those in other situations) were significantly more likely to engage in VPA ≥ twice a week. Children who strongly agreed with the fact that there were other children outside with whom they could play were also more likely to engage in VPA ≥ twice a week than children who strongly disagreed (OR: 1.80 [1.42–2.29]).

In the final logistic regression model, an interaction at the limit of significance (*p* = 0.059) was observed between gender and the presence of other children in the neighborhood with whom to play. Stratified analysis according to gender was therefore carried out (Table 4). Boys who strongly agreed with the fact that there were other children with whom to play were more likely to practice VPA ≥ twice a week than boys who strongly disagreed (OR: 1.93 [1.33–2.79]), while in girls, those who neither agreed nor disagreed were more likely to achieve the recommended level of VPA (OR: 2.18 [1.39–3.43]) (Table 4). Regarding the level of family affluence, girls in the “medium” and “low” groups were less likely to practice VPA ≥ twice a week than girls in the “high” group (Table 4). Among boys, this association was statistically significant only in the “medium” group. A significant association was observed with the family structure only in boys: boys who lived with both parents were more likely to achieve a recommended level of VPA than those in other family situations (Table 4). Finally, among girls, only age and perceived safety were significantly associated with VPA. When compared to girls aged 12–13, girls aged 10–11 who strongly agreed that the neighborhood was safe for walking and playing alone outside (compared to those who strongly disagreed) (OR: 1.66 [1.10–2.49]) were more likely to practice VPA ≥ twice a week (Table 4).

**Table 4:** Factors associated with the practice of vigorous physical activity (VPA) at least twice a week stratified by gender. HBSC, French-speaking Belgium, 2014

	Boys (*n* = 1004)				Girls (*n* = 936)			
	Adjusted OR (95%CI) Complete model	p	Adjusted OR (95%CI) Final model	p	Adjusted OR (95%CI) Complete model	p	Adjusted OR (95%CI) Final model	p
Age						0.03		0.02
10–11 years					1.43 (1.04–1.98)		1.48 (1.08–2.03)	
12–13 years					1		1	
Family structure		0.009		0.009		0.18		
Living with both parents	1		1		1			
Other family situations	0.64 (0.46–0.89)		0.64 (0.46–0.89)		0.81 (0.59–1.11)			
Family affluence scale		0.03		0.06		0.001		< 0.001
High	1		1		1		1	
Medium	0.65 (0.45–0.94)		0.65 (0.45–0.94)		0.65 (0.46–0.91)		0.63 (0.45–0.89)	
Low	0.66 (0.40–1.07)		0.66 (0.40–1.07)		0.45 (0.29–0.69)		0.42 (0.27–0.64)	
Size of the area where the school is located						0.32		
< 3000 inhabitants					1.32 (0.88–1.98)			
Between 3000 and 15,000 inhabitants					1.25 (0.89–1.75)			
> 15,000 inhabitants					1			
Safe neighborhood for walking or playing alone						0.049		0.049
Strongly agree					1.65 (1.09–2.48)		1.66 (1.10–2.49)	
Neither agree nor disagree					1.23 (0.85–1.79)		1.24 (0.86–1.80)	
Strongly disagree					1		1	
Presence of other children in the neighborhood for playing outdoors		0.001		0.001		0.004		0.003
Strongly agree	1.95 (1.34–2.82)		1.95 (1.34–2.82)		1.38 (0.97–1.95)		1.40 (0.99–1.98)	
Neither agree nor disagree	1.28 (0.79–2.08)		1.28 (0.79–2.08)		2.14 (1.36–3.37)		2.18 (1.39–3.43)	
Strongly disagree	1		1		1		1	

## Discussion

The main findings of this study were that, after adjustment, perceived security of the neighborhood (only for girls), presence of other children with whom to play outside in the neighborhood, and the existence of a playground or park were positively associated with physical activity. However, no association was found with the presence of a yard or garden at home.

Perceived neighborhood safety is a frequently used indicator in studies analyzing environmental factors associated with youth physical activity. This is a complex multidimensional concept. The two most frequently studied dimensions--and seemingly the most important concerns for young people and their parents--are road safety (traffic density, quality of public lighting, presence of bicycle lanes, etc.) and “stranger danger” (oral, physical or sexual violence, murder, etc.). [28]. In the literature, observations differ according to the dimension analyzed. Recent reviews concluded that perceived safety was positively associated with physical activity when road safety components are analyzed [13–15]. In terms of criminality, results on perceived security are mixed; at present, the relationship with physical activity is not clear.

Our results indicate that girls were more likely to practice VPA ≥ twice a week when they feel their neighborhood is safe. No association was observed for boys. One hypothesis is that girls are more concerned about safety than boys and that this fear negatively influences their outdoor physical activity. Some studies tend to support this hypothesis. An Australian study in children aged 10–12 found that girls who perceived local roads to be safe spent more time walking for transport and for exercise [29]. No association was observed for boys. Similarly, other studies reported that girls were more worried about strangers than boys [30–33]. They found a negative association between this fear and their level of physical activity.

Safety as perceived by parents should be taken into consideration. Parental concern for neighborhood safety appears to vary by child gender. Studies have shown that parents were likely to be more protective of daughters than sons [28, 34]. Similarly, a study published in 2016 found that parents perceived better “stranger” safety for boys [35]. Parental perception of safety is associated with that of their children and negatively affects the child’s physical activity [36, 17]. An American study based on data collected between 1999 and 2007 showed that both cross-sectional and longitudinal models indicated that children whose parents perceived their neighborhoods as unsafe had less physical activity. However, the magnitude of this association was much weaker in longitudinal models [37]. Results of a Canadian study showed that child-perceived safety was partly explained by parent-perceived safety, but seemed to be determined by distinct environmental features [38].

Regarding the presence of a playground or park in the neighborhood, our analysis showed that children living in a neighborhood with a playground or a park were more likely than others to reach the recommended threshold of GPA. This is consistent with the literature. Studies have shown that time spent outdoors was strongly associated with physical activity in children and adolescents [6]. The availability of outdoor playgrounds and parks is therefore likely to play an important role in children’s physical activity. Indeed, literature reviews focusing on environmental determinants of youth physical activity (ages 3 to 18) showed that, in most studies analyzed, the presence of neighborhood playgrounds or parks was positively associated with youth physical activity [13–16]. Comparative studies identifying the type of playground equipment most effective in improving physical activity are warranted.

In our study, children in neighborhoods where there were other children to play with outdoors were more likely to achieve a recommended level of GPA. No study exploring an association between this indicator and GPA has been found. However, one study focusing on time spent in active free play revealed that children with numerous friends in their neighborhood (as declared by parents) played more regularly on their own street than others [39]. Indeed, social support is frequently cited as being associated with higher levels of physical activity in children and adolescents [6]. Moreover, the sight of children playing may improve feelings of safety and encourage other children to do likewise. The importance of social links between neighbors (children and adults) in children’s outdoor physical activity has not been sufficiently explored and merits future research.

Our results also showed a positive association between the presence of other children to play with outside and VPA among boys only. In girls, the OR for the “strongly agree” category was very close to statistical significance (*P* = 0.06), underlying a loss of statistical power in stratified analysis. Furthermore, girls who neither agreed nor disagreed with the fact that there were other children in the neighborhood with whom to play outdoors were more likely to practice VPA ≥ twice a week. This result is difficult to interpret: some girls may have given this answer because they did not have permission to play outdoors in their neighborhood. The girls may have had difficulty answering this question because of the lack of nuance between the three response modalities.

Concerning the presence of a yard or garden at home for playing outside, no association was found in our study. This may be due to the high proportion of children with a garden or yard at home (89%). The lack of an observed association could also be explained by the fact that the indicator we used lacks precision. Indeed, it may be difficult to position oneself in relation to this indicator for children who lived, for example, in group dwellings where a yard or garden was shared. Instead, children may use other places to play or practice sports. It would thus be interesting to carry out research to identify the places where children are most active. A literature review focusing on home physical environment analyzed eight studies investigating the relationship between physical activity and the presence of a backyard [40]. One of these studies reported that 30–33% of physical activity took place in the yard [39]. Only one study out of five found a positive association between yard play equipment and outdoor physical activity [41]. In that study, the size of the backyard was also positively associated with outdoor physical activity.

As frequently reported in the literature, we found that boys were significantly more active than girls [6]. Concerning family structure, the stratified multivariate model for gender showed that boys living with both parents were more likely to practice VPA ≥ twice a week than boys in other family situations. Results of previous studies lack consistency, but two reviews concluded that the parental situation was unrelated to youth physical activity [42, 43]. Regarding socio-economic status, our results showed that the level of family affluence was positively associated with VPA, but not GPA. Results of reviews for the association between socio-economic status and physical activity are divergent [6, 42]. Similarly to our results, some studies showed that adolescents with high socio-economic status were more likely to engage in club sports than those with low socio-economic status [44, 45]. Furthermore, in our study, the proportion of children born abroad was higher in the low levels of family affluence than in the highest levels (13.3% versus 7.1%). A lower level of physical activity was thus expected among children born abroad, but the opposite was observed: children born abroad were more likely to achieve a recommended level of GPA than those born in Belgium. Nevertheless, in previous studies, immigrant status or having been born to immigrant parents seemed to be a disadvantage in achieving a recommended level of physical activity [46, 47]. The demographic and migratory background in Belgium may be involved in these contradictory conclusions; further research is needed to better understand such relationships.

One strength of our study lies in the fact that we analyzed indicators related to the child’s living environment, which had previously been only rarely studied. Our findings therefore provide new information for better understanding environmental factors possibly involved in youth physical activity. Moreover, we used a randomized sample as well as a large sample size. However, some limitations should be noted, one of which was the cross-sectional study design. Indeed, no causal link can be deduced from the observations made. In addition, no objective measure of physical activity or the child’s living environment was used. All indicators studied were based on self-reported data; thus, measurement bias is possible. However, children’s perception of their environment may also account for their taking part in physical activity. The proportion of children reporting VPA at least twice a week was high (76%). The wording of our questions may not have been sufficiently clear to some children to enable them to differentiate between MVPA and VPA; therefore, they might have incorporated both when answering the question related to VPA. Therefore, observed associations with VPA need to be interpreted with caution. Concerning environmental indicators, the modality of the response “neither agree nor disagree” posed difficulties in interpretation; it may refer to very different realities depending on the respondent. Nevertheless, it was decided to maintain it as such, since it could not be attached to either of the other two categories.

## Conclusion

Based on our findings and the literature, perception of the environment in which children live is likely to play an important role in their level of physical activity. Our results argue for developing actions aimed at creating a life environment more favorable to their daily physical activity. Improving neighborhood design by creating free-access playgrounds and by improving road safety could help children to become more active. Parental attitude, along with the presence of social networks in the neighborhood, might also play an important role in youth physical activity outdoors, but this warrants additional research. Indeed, children rely heavily on parental judgement and decisions, which may have a significant impact on their outside play time if the neighborhood is viewed by parents as unsafe. In contrast, development of social networks in neighborhoods could enable families to create links, and improve feelings of well-being and security. Further studies are needed to elucidate these questions and to identify interventions that would be most effective in making young people more active.

## Abbreviations

CI: confidence interval
GPA: global physical activity
HBSC: health behaviour in school-aged children
MVPA: moderate-to-vigorous physical activity
OR: odds ratio
VPA: vigorous physical activity
WHO: World Health Organization
